# MicroRNA let-7 Suppresses Influenza A Virus Infection by Targeting RPS16 and Enhancing Type I Interferon Response

**DOI:** 10.3389/fcimb.2022.904775

**Published:** 2022-07-07

**Authors:** Wenjiao Wu, Chao Wang, Changliang Xia, Shuwen Liu, Qinghua Mei

**Affiliations:** ^1^ Department of Pharmacy, Guangdong Second Provincial General Hospital, Guangzhou, China; ^2^ State Key Laboratory of Organ Failure Research, Guangdong Provincial Key Laboratory of New Drug Screening, School of Pharmaceutical Sciences, Southern Medical University, Guangzhou, China

**Keywords:** microRNA let-7, rps16, type I interferon, antivirals, influenza A virus

## Abstract

Given the frequent emergence of drug-resistant influenza virus strains and new highly pathogenic influenza virus strains, there is an urgent need to identify new antiviral drugs and targets. We found that influenza A virus (IAV) infection caused a significant decrease of microRNA let-7 expression in host cells; that overexpression of let-7 increased interferon expression and effectively inhibit IAV infection; and that let-7 targets the 3’-untranslated region (UTR) of the ribosomal protein 16 (*RPS16*) gene, decreasing its expression. Knocking down the expression of *RPS16* increased the expression of type I interferon and inhibited viral replication. The present study uncovered the regulatory effect of let-7b and let-7f on influenza A infection, which is a potential biomarker of IAV infection. In addition, let-7 may be a promising therapeutic agent against influenza A.

## Introduction

Influenza A virus (IAV) causes annual seasonal epidemics, leading to high morbidity and massive economic losses worldwide (Javanian et al., 2021). The World Health Organization has estimated that these epidemics result in approximately 4 million severe infection cases and approximately half a million deaths annually. Vaccination is the most effective prophylaxis against influenza infection. However, the high rate of IAV antigenic drift and shift hinders the efficacy of vaccination, creating a public health concern (Keshavarz et al., 2019). Two types of antivirals have been approved and recommended for use in chemoprophylaxis and treatment of influenza: (1) the neuraminidase inhibitors (NAIs) oseltamivir (Tamiflu), zanamivir (Relenza), and peramivir (Rapivab), and (2) the cap-dependent endonuclease inhibitor baloxavir marboxil (Xofluza). However, the administration of neuraminidase inhibitors is recommended within 48 h after the onset of symptoms (Eiland and Eiland, 2007; Wester and Shetty, 2016), and the occurrence of drug resistance affects NAI efficacy (McKimm-Breschkin, 2013; Tang et al., 2019). For baloxavir, except for its adverse effect (Locke et al., 2019), research on healthy subjects with influenza A and B revealed that approximately 10% exhibited a specific mutation (PA/I38X) after treatment, which has been related to higher viral loads and long-term symptoms (Uehara et al., 2020). Thus, more effective therapeutics need to be developed for prevention.

MicroRNAs (miRNAs) are a family of noncoding, single-stranded small RNA molecules, 18-22 nucleotides in length, regulate gene expression at the post-transcriptional level (Ambros, 2001; Ambros, 2004). Several studies have shown that miRNAs are key regulators in the pathology of viral infections (Skalsky and Cullen, 2010; Liao et al., 2021a). It has been shown that IAV infection induces the differential expression of host microRNAs, several of which have been found to inhibit replication by directly targeting IAV mRNAs. For example, it has been reported that miR-323, miR-491, and miR-654 decreased H1N1 replication by binding to the same conserved site on PB1 mRNA, consequently inducing PB1 mRNA degradation (Song et al., 2010). Many host miRNAs also indirectly exert regulatory functions. For example, many are involved in the antiviral immune response. A microRNAome study showed that the expression levels of 130 miRNAs significantly changed after the reconstruction of the 1918 influenza virus (r1918). Intriguingly, 18 of them target the host genes that are necessary for the extreme lethality of the r1918 virus, resulting in an increased IFN response or alteration of cell fate (Li et al., 2010). In contrast to the inhibition of H1N1 replication, a few miRNAs have been reported to facilitate virus replication. It has been shown that during early IAV infection, the virus induces the downregulation of miR-548 and subsequently increases the abundance of NS1ABP (Non-Structural-1A Binding Protein), a host protein that mediates apoptosis by facilitating viral replication (Othumpangat et al., 2013; Othumpangat et al., 2014). MicroRNA-21-3p has been shown to promote IAV replication, which was similar to the result of HDAC8 knockdown, indicating that miR-21-3p promotes IAV replication by suppressing HDAC8 expression (Xia et al., 2018). Notably, the roles of specific host miRNAs during influenza virus infections remain poorly defined, so miRNAs and their targets have become research foci for the development of specific gene drugs for the treatment of viral diseases (Hum et al., 2021).

In this study, using microarrays we identified the downregulation of two miRNA let-7 family members (hsa-let-7b and hsa-let-7f) during IAV infection, showing that they target RPS16 expression. Our findings suggest that let-7 and its target gene *RPS16* are potential targets for anti-influenza therapeutics.

## Materials and Methods

### Microarrays Informatics Analysis

Microarray series matrix files and platform information for GSE112728 and GSE107186 were downloaded from GEO (http://www.ncbi.nlm.nih.gov/geo), a public database of high-throughput gene expression data on the GPL24844 and GPL8179 platform, respectively. Data preprocessing included transformation of gene probes into gene symbols, data consolidation, and batch normalization. Probes lacking gene symbols or genes with more than one probe were deleted or averaged. Merged data were prepossessed using the SVA package in R software (version 4.0.0) to remove batch effects. The Limma package in R (version 4.0.0) software was utilized to screen DEGs between cell infected with/without H1N1 and H5N1 virus, followed by data normalization. Adjusted P values <0.05 and |log FC| (fold change) > 1 were considered statistically significant. The heatmap package in R software (version 4.0.0) was used to map DEG profiles.

### Cells and Virus

Human alveolar adenocarcinoma basal epithelial cell (A549) was cultured in RPMI1640 supplemented with 10% fetal bovine serum (FBS, Hyclone) and 1% penicillin-streptomycin at 37 °C in a humidified atmosphere of 5% CO_2_. Madin-Darby canine kidney (MDCK) cell, human bronchial epitherial mesothelial cell (BEAS-2B) and HEK-293T cells were cultured in DMEM supplemented with 10% FBS and 1% penicillin/streptomycin. The influenza virus A/WSN/1933 (H1N1) strain was cultured in 9-day-old embryonated eggs; virus-containing allantoic fluid was harvested and maintained at -80 °C for subsequent infection.

### IAV Infection

A549 cells (5×10^5^ per well) were seeded in six-well plates and incubated at 37 °C for 24 h. Prior to infection, the cells were washed twice with PBS (Phosphate Buffered Saline) and virus diluted in serum-free medium was added. After incubation for 1 h, cells were washed once with PBS, fresh serum-free RPMI1640 medium was added, and cells were further incubated for the indicated times at 37°C and 5% CO2.

### Plaque Assays

MDCK cells were seeded into 6-well plates (≈1.5×10^6^ per well) and cultured overnight until they grew into a monolayer. Equal volumes of diluted virus in serum-free medium were added to each well and the plates were incubated at 37°C for 60 1h with frequent shaking every 15 min. Then, a 1.2% Avicel overlay (3 ml) containing DMEM and trypsin (1.5 µg/ml) was added. Plates were incubated at 37°C in a humidified atmosphere of 5% CO2 for 48 h, cells were stained with 0.5% crystal violet for 30 min at room temperature, and plaques were counted.

### Transfection

The hsa-let-7b-3p and hsa-let-7f-3p mimics, mimic negative control (mimic NC), non-specific siRNA (siNC), and siRPS16 were purchased from RIBOBIO Biotechnology (Guangzhou, China). Transfection of miRNA mimics or siRNA was performed using Lipofectamine 2000 (Invitrogen, Thermo Fisher Scientific), according to the manufacturer’s instructions. At 48 h after transfection, cells were infected with IAV.

### Quantitative Reverse Transcription-PCR

Total RNA was isolated from cells using TRIzol reagent (Invitrogen, Carlsbad, CA, United States). It was reverse transcribed using the First-Strand cDNA Synthesis Kit (Promega, United States) with an oligo-dT primer for mRNA or a specific stem-loop primer for miRNA (Bulge-LoopTM miRNA qPCR primers, RiboBio, China). RT-PCR for miRNA and mRNA was performed using m the SYBR Green PCR kit (Promega, Madison, WI, USA) on a Loche Fast Real-Time PCR System. Relative quantification was performed by normalization to U6 (for miRNA) or GAPDH (for mRNA). Relative quantities were calculated using the 2^−ΔΔCT^ method. Primer sequences are provided in **Table 1**.

**Table 1:** Primer sequences for qRT-PCR.

Name	Primer sequences
HA- Forward	5’-TTCCCAAGATCCATCCGGCAA-3’
HA-Reverse	5’-CCTGCTCGAAGACAGCCACAACG-3’
RPS16-Forward	5’-TCTCATCAAGGTGAACGGGC-3’
RPS16-Reverse	5’-AAATCGCTCCTTGCCGAGAA-3’
IFN-β- Forward	5’ -ATGACCAACAAGTGTCTCCTCC-3’
IFN-β-Reverse	5’-GGAATCCAAGCAAGTTGTAGCTC -3’
IFN-α- Forward IFN-α-Reverse	5’-GCCTCGCCCTTTGCTTTACT-3’ 5’-CTGTGGGTCTCAGGGAGATCA-3’
IFITM3-Forward	5’-CATCCCAGTAACCCGACCG -3’
IFITM3-Reverse	5’- TGTTGAACAGGGACCAGACG-3’
Mx1- Forward	5’-GAGGTGGACCCCGAAGGA-3’
Mx1-Reverse	5’-CACCAGATCCGGCTTCGT-3’
GAPDH-Forward	5’-AGGGCAATGCCAGCCCCAGCG-3’
GAPDH-Reverse	5’-AGGCGTCGGAGGGCCCCCTC-3’

### Western Blotting

Total protein was isolated from cells using radioimmunoprecipitation assay (RIPA) buffer (Beyotime Biotechnology, China) with a protease inhibitor cocktail (Absin, China). Protein concentration was determined using a BCA protein assay kit (Solarbio Life Science, China). Proteins were separated by SDS-PAGE gel and then transferred onto polyvinylidene difluoride membranes (GE Healthcare, Freiburg, Germany). The membranes were blocked with 5% nonfat milk and incubated overnight at 4°C with primary antibodies against RPS16 (ab177951, Abcam, USA, 1:2000 dilution), NP (polyclonal ab128193, Abcam, USA, 1:2000), M2 (GTX125951, USA); IFNAR1 (ab124764, Abcam, USA, 1:1000), β-actin (3700s, Cell Signaling Technology, Danvers, MA, 1:2000) or GAPDH (#5174, Cell Signaling Technology, Danvers, MA, 1:2000) at 4 °C overnight. After incubation with secondary antibodies at room temperature, bands were detected using a chemiluminescence (ECL) kit (GE Healthcare).

### Dual Luciferase Activity Assay

The wild-type (wt) and mutant (mut) RPS16 3’-UTRs were PCR-amplified from A549 cDNA and cloned into the luciferase reporter vector pMIR-RB-ReportTM (Ambion). RPS16 3’-UTR target site mutations were constructed using a fast mutagenesis system (Transgen, China). The sequences of the RPS16 3′UTR construct used in this experiment were changed from 5’-TTGTATA-3’ to 5’-AACATAT-3’(as shown in **Figure 3A**). For validation of the RPS16 3’-UTR as a target of let-7b/f, cotransfections of 3’-UTR constructs (100ng/well), and let 7f mimic (or control mimic) (50nM) were carried out in HEK293T cells using Lipofectamine 2000. At 24h post transfection, cells were collected and luciferase activity was measured with a Dual-Luciferase assay (Promega, USA) according to the manufacturer’s protocol. Reporter gene expression activity was determined by normalizing the firefly luciferase activity to the Renilla luciferase activity.

### MicroRNA Pull-Down Assay

According to previous reports (Phatak and Donahue, 2017), the mimic of hsa-let-7b-3p was labeled with biotin at 5’ end. Seed the A549 cells (2×10^7^) in 10 cm tissue culture dish, then transfect the cells with control miRNA and 5’ biotin-labeled miRNA at a final concentration of 100 nM. Lipofectamine 2000 was used as a transfection reagent according to manufacturer’s guideline. Forty eight hours post transfection, the whole cell lysates were harvested with lysis buffer supplemented with protease inhibitor and RNase inhibitor on ice, centrifuge the lysates at 15,000 g for 10 min at 4 °C, and transfer the lysates to a new eppendorf tube. Aliquot 100µl cell lysate for input, the cell lysates were mixed with Streptavidin-Dyna beads (wash the beads with TES buffer before use) and rotated at room temperature for 1h. Wash the beads five times with 1 ml washing buffer. During every wash put the tube on a magnetic stand and remove the supernatant carefully without taking the beads out. Extract the RNA with TRizol reagent. Detect the hsa-let-7b binding RNA expression through RT-PCR. Primer and probe sequences are provided in **Table 2**

**Table 2:** Probe and primer sequences for miRNA pull-down assay.

Gene	Sequence	Label
hsa-let-7b-3p Probe	CUAUACAACCUACUGCCUUCCC	5’-biotin
Lac Z(NC) probe	CAAACGGCGGATTGACCGTAATGGGATAGGTCACGTTGGTGTAGATGGGCGCATCGTAAC	5’-biotin
RPS16 RT-PCR primer	F:5’-GCTCGCTACCAGAAATCCTAC-3’ R:5’-GCCACACACAGTTCTTGAAAC-3’	
hsa-let-7b-3p RT-PCR primer	5’-CTATACAACCTACTGCCTTCCC-3’	
U6 RT-PCR primer	F:5’-CTCGCTTCGGCAGCACA-3’ R:5’-AACGCTTCACGAATTTGCGT-3’	

### Click-iT Homopropargylglycine Protein Synthesis Assay

To detect the rate of protein synthesis, we utilized the lick-iT™ HPG Alexa Fluor™ 488 Protein Synthesis Assay Kit (Invitrogen, C10428). A549 cells were seeded onto 6-well plates with sterile coverslip on the bottom. The of mimics of let-7b/f (or miR NC control) and siRPS16 (or siNC control) (50 nM) were transfected into the cell, respectively. Protein synthesis was detected for cells transfected with let-7 mimics or siRPS16 for 48 h. Cells were treated with 50 μM HPG in L-methionine free medium for 1 h and fixed with 4% PFA in PBS. After permeabilization in 0.5% TritonX-100 in PBS, cells were incubated in Click-iT reaction cocktail at room temperature for 30 min in dark and then cell nuclei were counter stained with DAPI. Coverslips were removed from 6-well plates and mounted onto slides. The rate of HPG incorporation was examined through detecting the fluorescence intensity of cells at 488 nm excitation wavelength (Leica STELLARIS 8 FALCON). Images were analyzed using Image J for the HPG fluorescent intensity.

### Flow Cytometry

A549 cells were seeded onto 6-well plates with sterile coverslip on the bottom. The 50nM of mimics of let-7b/f (or miR NC control) and siRPS16 (or siNC control) were transfected into the cell, respectively. Flow cytometry was detected after the cells transfected for 48h. Cells were resuspended at the concentration of 5**×**10^5^/ml in 1**×** binding buffer at room temperature. Annexin V-FITC and PI (Propidium Iodide) were added to the flow cytometry tube containing 200 µl of the cells, mixed well and incubated in dark for 15 min according to the manufacture’s instruction. Cells were analyzed in an FACS analyzer (BD FACSAriaIII).

### IFN-β Antibody Neutralizing Assay

A549 cells were seeded onto 6-well plates and transfected with siRPS16 (or siNC control). After 48h of transfection, the cell was infected with influenza A/WSN/1933 (MOI = 0.1), the cells were maintained with medium containing IFN-β neutralizing antibody (2 µg/ml) (R&D system, MAB8141) or same concentration of antibody iso-type control IgG (R&D system, MAB004). The NP protein expression was tested by western blot assay at 24 h post infection.

### Statistical Analysis

GraphPad Prism 7 (GraphPad Inc., La Jolla, CA, U.S.A.) was used to process experimental data from triplicate experiments. Student’s *t*-test was used to compare data between two groups, and one-way analysis of variance (ANOVA) was utilized to compare multiple groups. *P*<0.05, *P*<0.01 and *P*<0.001 were considered statistically significant and denoted as *, **, and ***, respectively.

## Results

### Let-7 Expression Decreases After IAV Infection

**Figure 1:**
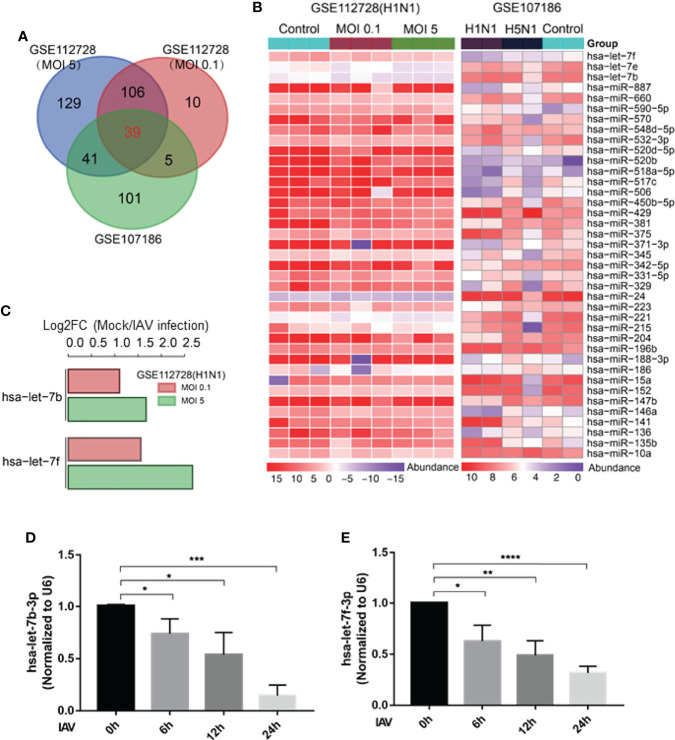
Let-7 expression decreases after IAV infection. **(A)** Venn diagram of differentially expressed miRNAs during IAV infection. **(B)** Heat map representing the 39 downregulated miRNAs from the two microarray dataset GSE112728 and GSE107186. **(C)** Analysis of hsa-let-7b and hsa-let-7f down-regulation of in GSE112728 microarray that the cell infected with 0.1 and 5 MOI of IAV compared with Mock cell. **(D, E)** A549 cell was infected with influenza A/WSN/1933 virus (MOI = 1) for 0, 6, 12 and 24h, the expression of hsa-let-7b-3p and hsa-let-7f-3p was accessed by real-time qPCR and normalized to expression of U6. Data are the mean ± SD from three independent experiments, *p < 0.05, **p < 0.01 vs. 0h control cell by *t*-test.

Various studies have shown that expression of cellular miRNAs is profoundly influenced by viral infection. The altered miRNA expressions lead to enhanced or suppressed antiviral responses, that may help in viral evasion or restrict virus infection. In this study, we reanalyzed the miRNAome sequencing data from GSE112728 and GSE107186 datasets. Data of GSE112728 was from a microarray that A549 cells were routinely infected with Influenza H1N1 (A/Memphis/14/96, A/WSN/33) for 12 hours with an MOI (multiple of infection) of 0.1 and 5. GSE107186 was a microarray that A549 cells were infected with A/Beijing/501/2009 (H1N1; MOI=5) and A/Vietnam/1194/2004 (H5N1; MOI = 2), for 24 or 48 h. The data were merged and re-analyzed, the differentially expressed miRNAs were identified using the cut-off criteria of absolute fold change ≥ 1 or ≤-1 and t-test P < 0.05 following H1N1 infection or H5N1 infection relative to mock-treated cells. As **Figures 1A, B** shown, there were 39 down-regulated genes in both two microarrays. Within the 39 down-regulated miRNAs (**Figure 1B**), three members of the miRNA lethal-7 (let-7) family (hsa-let-7b, hsa-let-7f, hsa-let-7e) were decreased. We validated the expression of let-7 *in vitro* by infecting A549 cells with influenza A/WSN/1933 for 0, 6, 12, and 24h. According to the result, let-7b-3p and let-7f-3p were gradually declined as the virus infection time progressed, that was consistent with the microarray results analysis (**Figures 1D, E**), the decrease of let-7e was not that obvious in our experiment (data not shown). We further analyzed the expression of hsa-let-7b and hsa-let-7f in the GSE112728 dataset, which the cells were infected with different doses of influenza A virus. As shown in **Figure 1C**, compared with mock, let-7b/f decreased along with the increased amount of viral infection. Thus, there was a down-regulation of has-let-7b and has-let-7f expression after cell infected with influenza A virus in lung epithelium cell, which intrigued our interest for further study.

### Let-7 Inhibits IAV Replication

**Figure 2:**
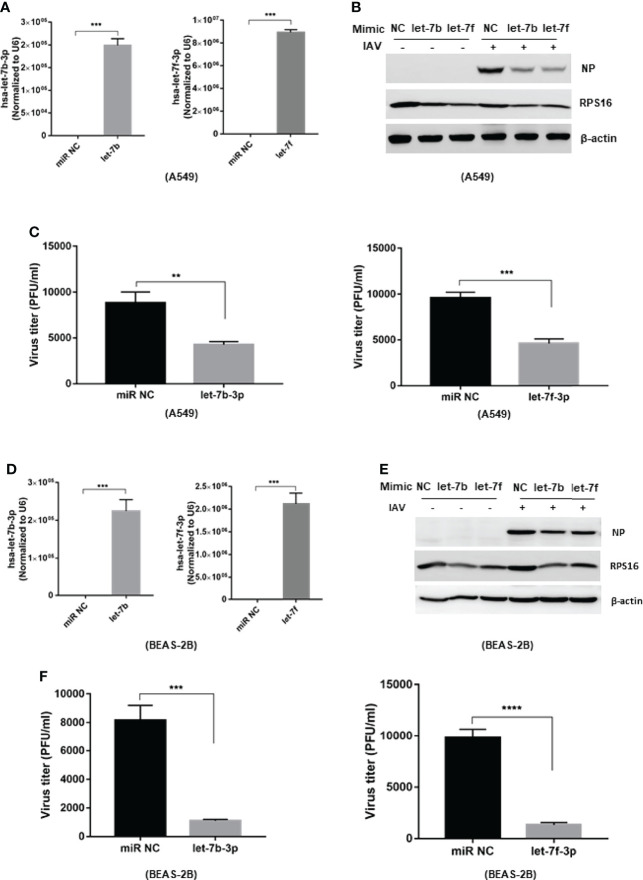
Over-expression of let-7b and let-7 inhibits influenza A virus infection to the host cell. The mimic of hsa-let-7b-3p and hsa-7f-3p (50nM) were transfected to A549 cell, the expression of let-7b-3p and let-7f-3p was detected by RT-PCR **(A)**. At 48h post transfection, the cells were infected with influenza A/WSN/1933 virus at 0.1 of MOI; **(B)** the influenza NP protein expression were measured by western blotting at 24h post infection; **(C)** the new born virus titer in the supernatants was tested by plaque forming assay and calculated. **(D)** The expression of let-7b-3p and let-7f-3p in BEAS-2B cell transfected with the mimic of hsa-let-7b-3p, hsa-7f-3p (50nM) for 48h. At 48h post transfection, the cells were infected with influenza A/WSN/1933 virus at 0.1 of MOI; **(E)** the influenza NP protein expression were measured by western blotting at 24h post infection; **(F)** the new born virus titer in the supernatants was tested by plaque forming assay and calculated. Data were shown as mean ± SD (**p < 0.01, ***p < 0.001).

To study the regulatory role of let-7b/f in influenza A virus infection, we transfected has-let-7b-3p and hsa-let-7f-3p mimics into A549 cells (**Figure 2A**). A549 cells were infected with influenza A/WSN/1933 (MOI=0.1) at 48h after transfection. Viral protein expression was analyzed by western blot at 24 h post infection. As the data shown, the expression of influenza NP protein in host A549 cells was reduced in cells transfected with let-7b-3p and let-7f-3p mimics (**Figure 2B**). Besides, the RT-PCR showed that both mimics decreased viral HA gene expression in host cells compared to miR NC control (**Supplementary Figure 2A**). To further confirm the role of let-7b and let-7f in IAV replication, we tested the functional new born virus titer in culture supernatants using plaque forming assays. As the data showed, both let-7 mimics decreased viral titers about 2~3 folds (**Figure 2C**). Besides, we transfected let-7 mimics (or control miR NC) in BEAS-2B cell (**Figure 2D**) and infected the cell with influenza A/WSN/1933 (MOI=0.1) at 48h after transfection, the viral NP was detected by western blot assay and the virus titer in the cell cultured supernatants were determined by plaque forming assay, the result was consistent with that in A549 cell (**Figures 2E, F**). These results revealed the antiviral potential of let-7b and let-7f mimics against IAV infection.

### *RPS16* Is a Direct Target of let-7

**Figure 3:**
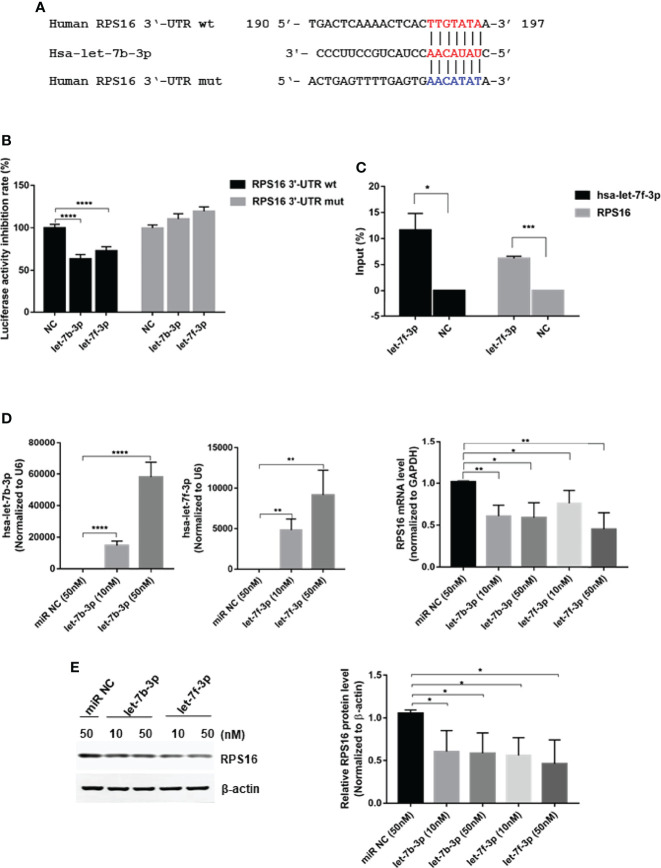
RPS16 is a direct target of let-7. **(A)** Schematic representation of predicted target sites of hsa-let-7b-3p in the 3’-UTR of *RPS16* and mutant RPS16 3’-UTR reporter constructs. **(B)** 293T cells were co-transfected with RPS16-3’-UTR luciferase reporter plasmid, RPS16-3’-UTR mut luciferase reporter plasmid (100ng/well), together with negative mimic (miR NC) or let-7b-3p/let-7f-3p mimic (50 nM). After 48 h, firefly luciferase activity was measured and normalized to Renilla luciferase activity. Data are the mean ± SD from four independent experiments. (****P < 0.0001 vs. negative mimic by *t*-test. **(C)** RNA pulldown assay indicated that biotin−hsa-let-7b-3p precipitated with RPS16 mRNA, Lac Z(NC)served as a negative control, the precipitated mRNA was reverse transcripted into cDNA and detected by RT-PCR, the relative expression level was compared to the gene expression in the input RNA. Data were shown as mean ± SD, *p < 0.05, ***p < 0.001. **(D)** A549 cells were transfected with let-7b-3p/let-7f-3p mimic or negative mimic (miR NC) at a final concentration of 10 nM and 50 nM, respectively. After 24 h, the expression of RPS16 was measured by real-time qPCR and normalized to expression of GAPDH. Data are the mean ± SD from three independent experiments. *p < 0.05, **p < 0.01 vs. negative control by t-test. **(E)** A549 cells were transfected with let-7b-3p/let-7f-3p mimic or negative mimic (miR NC) at a final concentration of 10 and 50 nM, respectively. After 48 h, RPS16 protein expression was analyzed by Western blot and the gray scale quantitation of the bands were analyzed by Image J software and normalized to the loading control. β-actin was used as loading control. Data are representative of three independent experiments, *p < 0.05.

To regulate viral replication, miRNAs may target host genes required for the viral life cycle or viral genes by binding to their 3’-UTRs. Target sites for let-7b and let-7f were predicted using two algorithm-based programs: TargetScan version 6.2 (http://www.targetscan.org) and MicroCosm (version 5.0, http://www.ebi.ac.uk/Enright-srv/microcosm/cgibin/targets/v5/search.pl). We found the predicted target sites for let-7b and let-7f in the 3’-UTR of the human *RPS16* gene (**Figure 3A**), which belongs to the ribosomal proteins family playing important role in virus infection (Wan et al., 2007; Han et al., 2020). To confirm the binding and function of let-7b and let-7f on RPS16, wild-type (wt) and mutant (mut) RPS16 3’-UTRs were PCR-amplified from A549 cDNA and cloned into the luciferase reporter vector pMIR-RB-ReportTM. The sequences of the RPS16 3’-UTR construct used in this experiment were changed from 5’-TTGTATA-3’ to 5’-AACATAT-3’(as shown in **Figure 3A**). For validation of the RPS16 3’-UTR as a target of let-7b/f, cotransfections of 3’-UTR constructs (100ng/well) and let 7f mimic (or control mimic) (50nM) were carried out in HEK293 cells using lipofectamine 2000. At 24h post transfection, cell lysates were collected and luciferase activity was measured with a Dual-Luciferase assay (Promega, USA) according to the manufacturer’s protocol. The result showed that let-7b-3p and let-7f-3p inhibited the luciferase activity of 3’-UTR of *RPS16* gene, whereas the inhibitory effects were ruined by the mutation of the binding sequence. Furthermore, a RNA pull-down assay was performed to test the binding of let-7 with *RPS16* mRNA. The results indicated that biotin labeled hsa-let-7b-3p showed obvious precipitation with host *RPS16* mRNA in A549 cell compared with the Lac Z(NC)control (**Figure 3C**), suggesting the direct interaction between let-7b−3p and *RPS16* mRNA. To further confirm the regulatory effect of let-7 on *RPS16* expression, we measured *RPS16* expression both at mRNA (**Figure 3D**) and protein (**Figure 3E**) levels after transfection of the let-7b/f mimics with different dosage (10nM and 50nM); as shown in **Figure 3D**, transfection of let-7b-3p mimic at 10 nM and 50 nM both inhibited RPS16 mRNA and protein expression compared with miR NC. However, we found that the dosage response of let-7b-3p mimic was not that obvious both at mRNA and protein level. Possibly under our experimental conditions, the inhibitory effect of let-7b-3p mimic on RPS16 reached the maximum. Also, when the cell was transfected with let-7f-3p mimic, the expression of RPS16 was both inhibited at gene and protein level; the expression of RPS16 was reduced by the increase level of let-7f expression (**Figures 3D, E**, **2B, E**). Based on our results, RPS16 should be one of the host targets of let-7b/f.

### RPS16 Promotes IAV Replication

**Figure 4:**
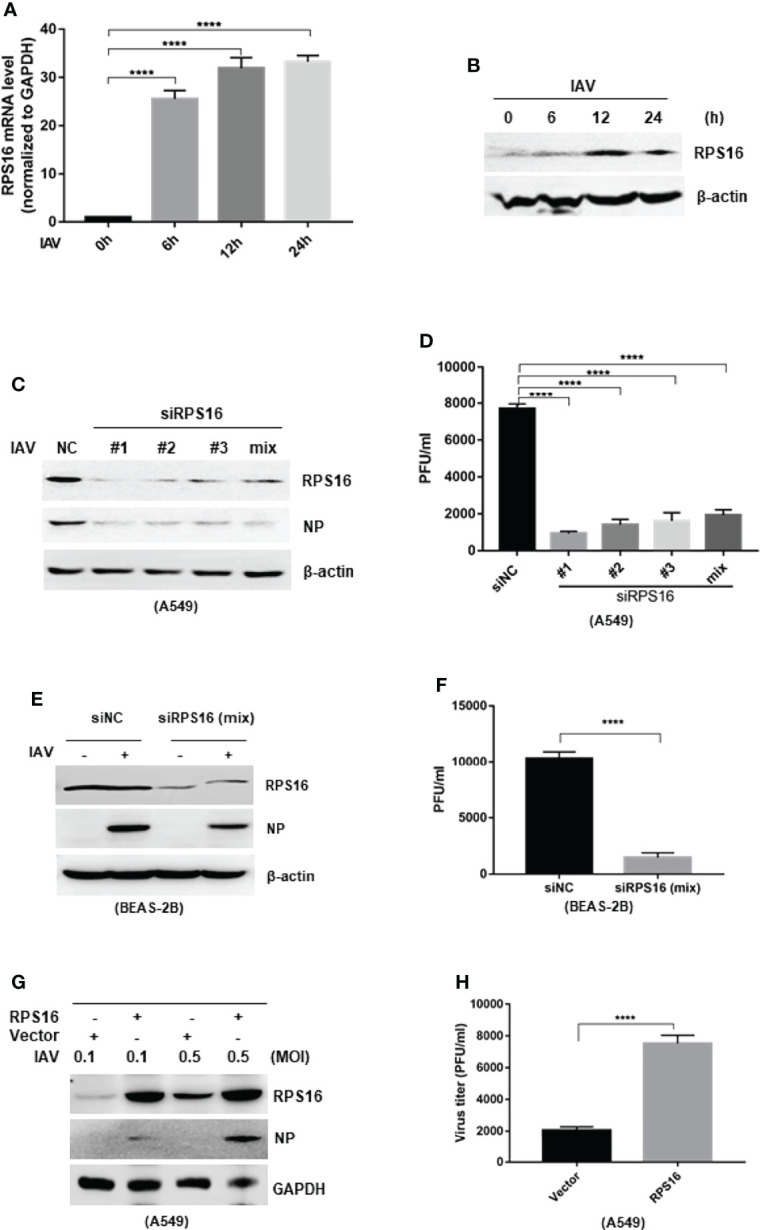
RPS16 promotes IAV replication. A549 cell was infection with influenza A/wsn/1933 virus (MOI=1) for 0, 6, 12 and 24h; **(A)** the expression of RPS16 mRNA was detected by real-time qPCR and normalized to expression of GAPDH. Data are the mean ± SD from three independent experiments, ****P < 0.0001 vs. 0h control cell by *t*-test; **(B)** the expression of RPS16 protein after IAV infection was detected by western blot. Data are representative of at least three independent experiments. (C and D) A549 cells were transfected with siNC or siRPS16. After 48 h, the cells were infected with influenza A/wsn/1933 virus of 0.1MOI, the expression of influenza NP protein was detected by westen blot **(C)**; **(D)** The virus titer in the supernatants was infected to MDCK cell and measured the viral titer by plaque forming assay, data were shown as mean ± SD (**p < 0.01, ***p < 0.001). (E and F) BEAS-2B cells were transfected with siNC or siRPS16. After 48 h, the cells were infected with influenza A/wsn/1933 virus (MOI = 0.1), the expression of influenza NP protein was detected by westen blot **(E)**. The virus in the supernatants was infected to MDCK cell and measured the viral titer by plaque forming assay **(F)**, data were shown as mean ± SD (**p < 0.01, ***p < 0.001). **(G, H)** A549 cells were transfected with RPS16 expressing vector or control vector. After 48 h, the transfected cells were infected with influenza A/wsn/1933 virus of 0.1 MOI and 0.5 MOI, the expression of influenza NP protein was detected by westen blot **(E)**; the virus in the supernatants infected with 0.1 MOI was measured the viral titer by plaque forming assay **(F)**. Data were shown as mean ± SD (**p < 0.01, ***p < 0.001). Data are the representative of three independent experiments.

As ribosomal proteins were found to play important role in virus infection, we wondered whether the inhibitory effect of IAV protein expression and replication by let-7b/f is associated with RPS16. Firstly, we checked the regulation of RPS16 in influenza A virus infection. As previous experiment, A549 cell was infected with influenza A/WSN/1933 at 1 MOI, then test the expression of *RPS16* at mRNA and protein levels at 0, 6, 12, and 24 h post infection by qRT-PCR and western blotting, respectively. As the data shown, contrary to the decreased let-7 expression, RPS16 increased at both mRNA transcription level and protein level (**Figures 4A, B**), indicating that RPS16 presents a putative antiviral target for IAV. Furthermore, when silenced the expression of RPS16 in A549 cells, the expression of influenza NP was detected by western blot and the virus titer was determined by plaque forming assay. As the data revealed, the influenza NP expression in the cell was inhibited by siRPS16 transfected; at the same time, the new born virus titer was reduced by 3~4 folds after siRPS16 transfection compared with siNC transfected cell (**Figures 4C, D**). Also, we got the similar result after transfecting siRPS16 into BEAS-2B cell and infecting with IAV (**Figures 4E, F**). In contrast, the exogenous expression of RPS16 in A549 cells increased viral NP expression and new born virus titer (**Figures 4G, H**). The above data determined the pro-viral effect of RPS16 for influenza A virus.

### Overexpression of let-7 or Knockdown of RPS16 Does not Affect Cellular Protein Synthesis and Cell Viability

Considering RPS16 is a ribosomal protein and may play a regulatory role in the global protein translation, we wondered whether the protein translation level was affected in the cell transfected with let-7 mimics/siRPS16. Protein synthesis was detected for cells transfected with let-7 mimics or siRPS16 through HPG protein synthesis assay. After transfection, cells were incubated with 50 μM HPG in L-methionine free DMEM media according to the manufacture’s instruction, cell nuclei were counter stained with DAPI. The rate of HPG incorporation was examined by the fluorescent intensity at the excitation wavelength of 488 nm. According to results, there was no significant difference in the immunofluorescence level between siNC and siRPS16 (**Supplementary Figures 1A, B**). At the same time, intracellular overexpression of let-7b/f did not significantly inhibit the overall protein expression translation level (**Supplementary Figures 1A, B**). Thus, the down-regulation of RPS16 does not effect on global protein translation in the cell.

Besides, we detected the cytotoxicity induced by let-7 mimics and siRPS16 transfection. The flow cytometry was conducted after the cell was transfected with let-7b/f mimic (or miR NC control) and siRPS16 (or siNC control) for 48h, respectively. Then the cells were staining with Annexin-V/PI and analyzed by flow cytometer. The result showed that the Annexin-V positive and PI positive cell populations were similar in each groups (**Supplementary Figures 1C, D**). The PI staining is one of the method usually used to detect the cell death, as **Supplementary Figure 1D** showed, the percentage of cell transfected with let-7b and let-7f mimic was about 84.35% and 83.35%, respectively; which showed no significance compared with miR NC control transfected cell (83.18%). In the cell transfected with siRPS16 RNA, the percentage of live cell is 87.87%, there was no difference compared with siNC transfected cell (88.68%). Thus, overexpression of let-7b/f or knockdown of RPS16 did not cause obvious cytotoxicity.

### Let-7 Upregulates IAV-Triggered Type I IFN Induction

**Figure 5:**
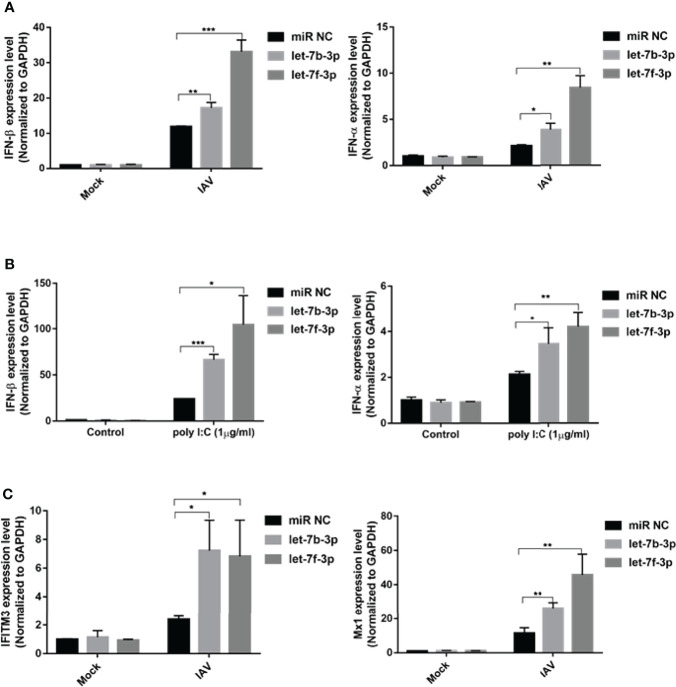
Let-7 upregulates IAV-triggered type I IFN induction. A549 cell was transfected with the mimics of hsa-let-7b-3p, hsa-7f-3p or negative control (miR NC). **(A)** At 48h post transfection, the cells were infected with influenza A/WSN/1933 virus (MOI=1) for 12h, the expression of IFN-β and IFN-α were detected by real-time qPCR and normalized to expression of GAPDH; **(B)** At 48h post transfection, the cells were transfected with poly I:C (1µg/ml) for 12h, the expression of IFN-β and IFN-α were detected by real-time qPCR and normalized to expression of GAPDH. **(C)** At 48h post transfection, the cells were infected with influenza A/WSN/1933 virus at 1 of MOI for 12h, the expression of IFITM3 and Mx1 were detected by real-time qPCR and normalized to expression of GAPDH. Data were shown as mean ± SD (*p < 0.05, **p < 0.01, ***p < 0.001). Data are the representative of three independent experiments.

**Figure 6:**
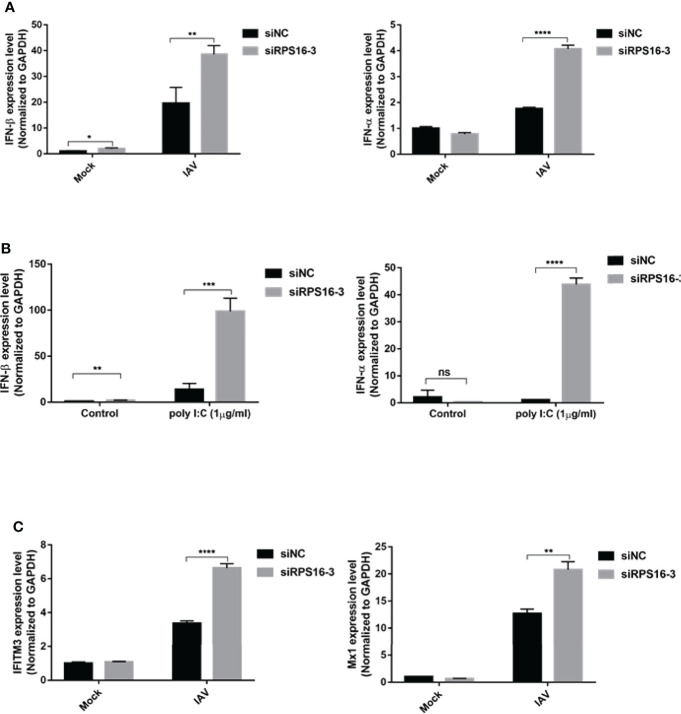
RPS16 is a type I IFN repressor during virus infection. A549 cell was transfected with siRNA specific to RPS16 (siRPS16) or negative control (siNC). **(A)** At 48h post transfection, the cells were infected with influenza A/WSN/1933 virus at 1 of MOI for 12h, the expression of IFN-β and IFN-α were detected by real-time qPCR and normalized to expression of GAPDH; **(B)** At 48h post transfection, the cells were transfected with poly I:C (1µg/ml) for 12h, the expression of IFN-β and IFN-α were detected by real-time qPCR and normalized to expression of GAPDH. **(C)** At 48h post transfection, the cells were infected with influenza A/WSN/1933 virus at 1 of MOI for 12h, the expression of IFITM3 and Mx1 were detected by real-time qPCR and normalized to expression of GAPDH. Data were shown as mean ± SD (*p < 0.05, **p < 0.01, ***p < 0.001). Data are the representative of three independent experiments.

IFN-β is the primary antiviral cytokine during early IAV infection. Once released, type I IFNs bind to the IFNAR1 receptor on target cells, which activates the Jak-STAT signaling pathway to induce the transcription of multiple IFN-stimulated genes (ISGs) (Mazewski et al., 2020). To determine whether the antiviral effects of let-7b and let-7f were associated with IFN-I induction, we checked IFN-β and downstream ISGs, including Mx1 and IFITM3 expression, using qRT-PCR. Both let-7b-3p and let-7f-3p mimics upregulated the expression of IFN-β and IFN-α after IAV infection (**Figure 5A**). To test whether let-7b/7f also play a similar regulatory role in other RNA virus infections, we further transfected RNA virus nucleic acid analogs (poly I:C) in cells with/without the let-7b/f overexpression and detected the expression of type I interferon after poly I:C transfection, the results demonstrated the broad-spectrum regulation of virus infection by miRNA let-7 (**Figure 5B**). The IFN-Is downstream ISGs including IFITM3 and Mx1 were upregulated under let-7b-3p/let-7f-3p mimic treatment (**Figure 5C**). The role of RPS16 in type I IFNs induction was verified using RNAi. Within three of siRPS16 RNA, cell transfected with siRPS16-3 was the most obvious and consistent for regulating IFN-α/β, so we chose siPRS16-3 for the mechanism study. As shown in **Figures 6A, B**, the expression of IFN-β and IFN-α was significantly increased in the siRPS16-treated cells than in siNC control cells after IAV infection and poly I:C transfection, ISGs including IFITM3 and Mx1 were upregulated under siRPS16 transfection during IAV infection. Our data indicate that the regulatory effects of miRNA let-7b, let-7f, and their downstream target *RPS16* might be associated with the induction of a type I interferon antiviral response in the host cell. We also detected the inflammatory cytokines including IL-6 and TNF-α which play important roles in viral infection caused tissue damage and cytokine storm after the infected cell were treated with let-7b/f mimics. The result showed that the expression of IL-6 and TNF-α were lower or comparable in cell treated with let-7b/f mimics than the untreated control cell (**Supplementary Figure 2C**). The result determined that the miRNA let-7b/f could increase the antiviral IFN-Is without inducing excessive inflammatory response.

### Dampening Type I Interferon Signaling Impairs Inhibition of IAV Replication by let-7 and siRPS16

**Figure 7:**
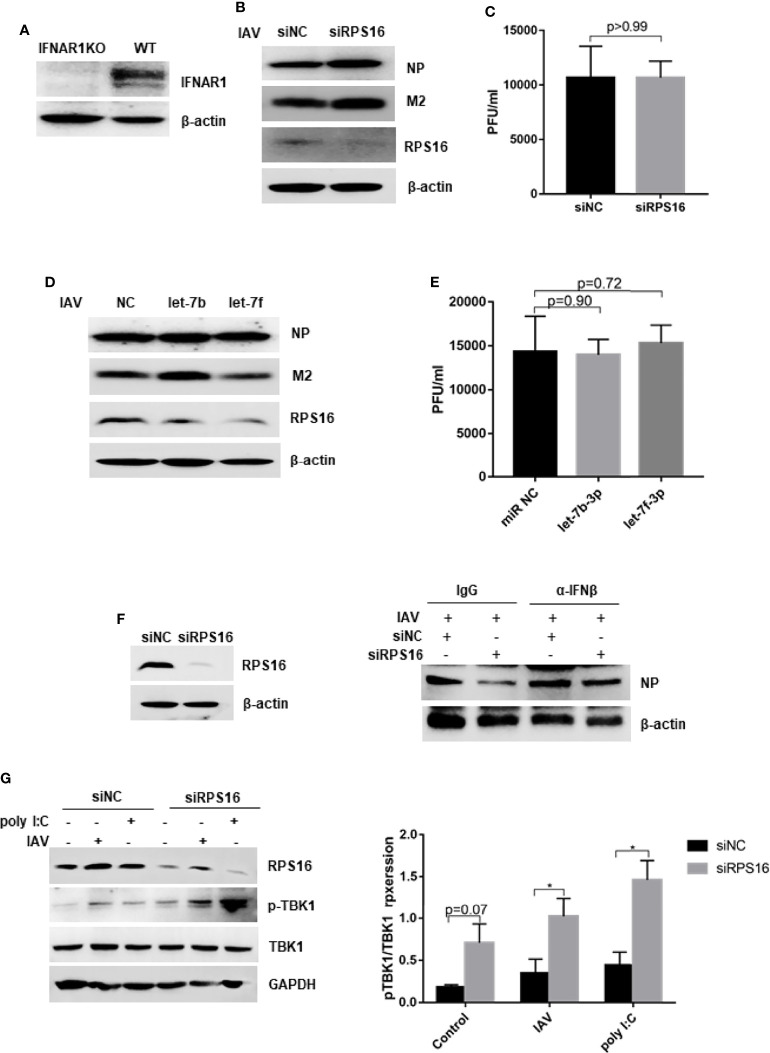
Dampening type I IFN signaling impairs inhibition of IAV replication by let-7 and siRPS16. **(A)** The expression of IFNAR1 was tested by western blot in IFNAR knock out (KO) and wild type A549 cell. The IFNAR1-/- A549 cell was transfected with 50nM mimics of hsa-let-7b-3p, hsa-7f-3p or negative control (miR NC); **(B)** at 48 h post transfection, the cells were infected with influenza A/WSN/1933 virus (MOI = 0.1), the expression of influenza NP and M2 protein were detected by western blot; **(C)** the new born virus titer in the supernatants was tested by plaque forming assay. IFNAR1-/- A549 cell was transfected with siRPS16 or siNC (50nM); at 48h post transfection, the cells were infected with influenza A/WSN/1933 virus (MOI = 0.1), **(D)** the expression of influenza NP and M2 protein were detected by western blot; **(E)** the new born virus titer in the supernatants was tested by plaque forming assay; **(F)** wild-type A549 cell was transfected with siRPS16 (or siNC control), the transfected cell was infected with influenza A/WSN/1933 virus (MOI = 0.1) at 48h post transfection; the cell was maintained with the medium including IFN-β or isotype control IgG. Viral NP expression was detected by western blot at 24 post infection. **(G)** Wild-type A549 cell was transfected with siRPS16 or siNC. At 48h post infection, the cell was infected with 3 MOI of influenza A/WSN/1933 virus or transfected with 1µg/ml poly I:C for 12h, the expression of TBK1 was detected by western blot. The gray scale of pTBK1 was analyzed by Image J normalized to the gray scale intensity of TBK1. Data are the representative of three independent experiments.

To determine whether the inhibitory effects of let-7b, let-7f, and siRPS16 were related to the type I interferon-mediated antiviral response, we measured virus replication in *IFNAR1*-deficient A549 (IFNAR1 KO) cells (**Figure 7A**). We infected with influenza A/WSN/1933 virus at an MOI of 0.1 in the presence of the let-7b-3p and let-7f-3p mimics, or the control miR NC mimic. At 24 h post infection, the expression of influenza NP/M2 protein and virus titer in cells treated with control mimic were comparable to that in cells transfected with the let-7b-3p/let-7f-3p mimics (**Figures 7B, C**). We observed the similar results in cells transfected with siRPS16 (**Figures 7D, E**). IFN-β neutralizing assay was conducted to test whether the regulatory effect of let-7-RPS16 on viral replication could be blocked by IFN-β neutralizing antibodies. We infected siRPS16 (or NC control) transfected cell with IAV for 1 h, then the cells were maintained with culture medium containing IFN-β or the same antibody isotype (IgG) control, respectively. The expression level of NP protein was detected 24 h post infection. The results showed that the antiviral effects of siRPS16 was weakened when IFN-β neutralizing antibody. Therefore, our experiments once again verified that the regulatory effect of let7-RPS16 on the virus is partially related to the antiviral effect mediated by type I interferon (**Figure 7F**). According to our results, RPS16 might be a critical regulator for IFN-Is antiviral signaling pathway. Studies have shown that in the antiviral response signaling pathway of type I interferon, the deubiquitinase USP1 interacts with TBK1 to deubiquitinate TBK1, enhance the expression of downstream type I interferon, and inhibits viral replication (Yu et al., 2017). A previous study found an interaction between RPS16 and USP1 in hepatoma cells (Liao et al., 2021b). Also, our result showed that knockdown of RPS16 in the cell obviously increased the phosphorylation of TBK1 (**Figure 7G**). Thus, our study showed that let-7 might inhibit the expression of RPS16 to inhibit IAV infection by promoting the type I interferon antiviral response.

## Discussion

The expression of many miRNAs in humans and animals is triggered in response to environmental stimuli, including viral infection. Recent studies have shown that miRNAs play critical roles in viral replication and survival. Next-generation sequencing (NGS, or deep sequencing) is a powerful tool to identify differentially expressed miRNAs, especially those with low abundance, under physiological perturbations. However, there was little commonality or difference in the direction of regulation among the affected miRNAs during IAV infection from the microarray data. Several potential reasons may account for the differences between these studies, such as host genetic background, IAV subtype, and heterogeneous endogenous tissues. In this study, we reanalyzed the data from two miRNA microarray databases to identify differentially expressed genes after IAV infection. Most published miRNA studies have focused on upregulated miRNAs; in this study, we focused on downregulated miRNAs to identify those associated with IAV infection. We found that the expression of two members of the miRNA let-7 family: let-7b and let-7f, was downregulated by IAV infection (**Figures 1A–C**). A previous study reported that let-7c inhibits IAV replication by directly binding to the 3’-UTR of the viral PB2 gene (Ma et al., 2012). The regulatory functions of the other two members were still unknown. Fortunately, we found a gradual decrease of hsa-let-7b-3p and hsa-let-7f-3p expression over the course of IAV infection in A549 cells (**Figures 1D, E**). Moreover, when cells were transfected with miRNA mimics of let-7b-3p and let-7f-3p, IAV replication was efficiently repressed (**Figure 2**). Therefore, in this study, we attempted to reveal the mechanism of miRNAs let-7b and let-7f in IAV infection. Our study provides a better understanding of the interaction between IAV and host cells; at the same time, it has identified potential antiviral miRNA therapeutics.

To regulate viral replication, miRNAs may target host genes that are required for the viral life cycle or viral genes. Ribosomal proteins (RPs) are important components of ribosomes that have important structural and regulatory roles in protein synthesis. It has been shown that RPs interact with non-ribosomal components in cells to exert effects not directly related to ribosomal function (Zhou et al., 2015). Ribosomal protein S3 (RPS3), a subunit of NF-κB, binds to RelA (p65), enhances its DNA-binding activity, and promotes the transcription and expression of downstream cytokines of NF-κB (Wan et al., 2007). Knockdown of *RPS27* blocked the phosphorylation of p65 protein S536 and IκBα protein S3, inhibiting the entry of NF-κB into the nucleus and reducing its DNA-binding activity, thereby blocking the NF-κB signaling pathway (Wan et al., 2011). RPL13 (Ribosomal Protein L13), as a critical regulator of IRES-driven translation of foot-and-mouth disease virus (FMDV) but found that it is not essential for cellular global translation, is also a determinant for translation and infection of Seneca Valley virus (SVV) and classical swine fever virus (CSFV) (Han et al., 2020). Study have also found that RPL13 interacted with retinoic acid-inducible gene-I (RIG-I) and binds to the 3’-UTR of NF-κB mRNA to promote NF-κB activation and downstream inflammatory gene expression (Zhang et al., 2013). It has been shown that RPs are abnormally expressed in a variety of tumor cells and are involved in tumor initiation, development, and metastasis (Ebright et al., 2020). Recent studies have shown that RPs play key regulatory roles in viral infections (Li, 2019). Primary regulatory mechanisms include: (a) binding to the virus-dependent internal ribosome entry site (IRES) and ribosomes (Hertz et al., 2013); (b) direct interaction with viral proteins (Cervantes-Salazar et al., 2015); and (c) regulation of the host immune response (Guan et al., 2021). We found that hsa-let-7b and hsa-let-7f bind to the 3’-UTR of one the gene encoding the ribosomal protein RPS16 (**Figure 3A**). The mimics of hsa-let-7b and hsa-let-7f showed an inhibitory effect on the luciferase activity of the report vector linked to the RPS16 3’-UTR (**Figure 3B**). Furthermore, the miRNA pull-down assay confirmed the direct binding of let-7 with the mRNA of RPS16 (**Figure 3C**). In addition, overexpression of let-7b and let-7f could repress RPS16 expression at both the mRNA and protein levels (**Figures 3D, E**). Thus, we speculated RPS16 could be one of the host targets of let-7.

Does RPS16 paly an important role in influenza A virus infection? The results showed that when we knock down the expression of RPS16 in host A549 and BEAS-2B cell, the replication of influenza was inhibited; exogenous expression of RPS16 increased virus replication (**Figure 4**). In contrast to let-7 expression, RPS16 was up-regulated at both the gene and protein levels when the host cells were infected with influenza virus (**Figures 4A, B**). At the same time, we also detected the inhibition of RPS16 protein expression when let-7-overexpressing cells were infected with/without influenza A virus (**Figures 2B, E**). Therefore, we speculate that let-7 may regulate influenza A virus infection of host cells through RPS16.Thus, we hypothesized that let-7b and let-7f inhibit IAV infection by repressing RPS16 expression.

Innate immunity is the primary barrier to pathogen invasion. After viral infection, the innate immune system recognizes multiple pattern-recognition receptors and triggers downstream signal transduction, leading to the production of cytokines, particularly IFN-α and IFN-β. Both of these IFNs bind to their cognate receptors on target cells, activating the Jak-STAT signaling pathway to induce transcription of IFN-stimulated genes (ISGs) (Mazewski et al., 2020). Recent studies have shown that miRNAs can regulate the replication of several viruses by regulating the production of IFNs and ISGs. Viruses have evolved multiple mechanisms to evade the IFN system and establish a productive infection. Typically, during infection, viral proteins inhibit either pattern-recognition receptor recognition or downstream signaling cascades to prevent ISG induction. However, a role for virus-mediated regulation of the IFN signaling cascade through changes in host miRNA levels has recently been proposed. For example, Japanese encephalitis virus (JEV) induction of miR-146a in infected cells has been shown to negatively regulate the IFN signaling protein tumor necrosis factor receptor-associated protein 6 (TRAF6), thus preventing the induction of IFN-α/β (Deng et al., 2017). The induction of miR-29c in influenza-infected cells has also been found to limit cytokine and chemokine responses by inhibiting various antiviral proteins and preventing overactive IFN and inflammatory responses (Pang et al., 2018). In our study, we found that when cells were transfected with let-7 mimics, the expression of type I interferon and ISGs after IAV infection was upregulated (**Figure 5**). In addition, the expression of type I interferon is upregulated after influenza virus infection when RPS16 is knocked down in cells (**Figure 6**). At the end of our study, we measured IAV replication in the presence of miRNC or let-7 mimics and transfected with siNC or siRPS16 in the type I interferon receptor 1 (IFNAR1) knockout cells, finding no significant difference in influenza virus NP protein and virus titers, which indicated that the inhibitory effects of the let-7 mimic and siRPS16 on influenza virus replication were attenuated (**Figures 7A–E**). Thus, let-7 is a potential anti-influenza therapeutic agent that inhibits influenza virus replication by regulating the induction of the type I interferon-induced antiviral response.

Furtherly, when the RPS16 knockdown cell were treated with IFN-β neutralizing antibody after influenza A virus infection, the expression of influenza NP were increased compared with the cell treated with IgG control, indicating the increased virus replication in the cell (**Figure 7F**). Thus, the antnviral effect of siRPS16 might be associated with type I interferon response. TBK1 is the most critical protein kinase in the synthesis of type I interferons and plays an important role in the antiviral innate immune response. It is an important node protein, and its activation is complicated and precise through a series of mechanisms, including phosphorylation, ubiquitination, and SUMOylation. Studiy have shown that in the antiviral response signaling pathway of type I interferon, the deubiquitinase USP1 interacts with TBK1 to deubiquitinate TBK1, enhance the expression of downstream type I interferon, and inhibits viral replication (Yu et al., 2017). A previous study found an interaction between RPS16 and USP1 in hepatoma cells (Liao et al., 2021b). Also, our result showed that knockdown of RPS16 in the cell obviously increased the phosphorylation of TBK1 (**Figure 7G**). Therefore, we speculated that TBK1 might be a key downstream factor for RPS16 to play a regulatory role. However, is there a direct interaction between RPS16 and TBK1, or whether it plays a regulatory role through other key factors such as USP1, we will further explore in subsequent studies.

A20 is an endogenous negative regulator of NF-κB signaling, which has been widely described in several autoimmune and inflammatory disorders and more recently in terms of chronic lung disorders. (Momtazi et al., 2019). Maelfait et al. and his team have demonstrated A20 (Tnfaip3) is a negative regulator of Toll like receptor (TLR) in rheumatoid arthritis (Matmati et al., 2011). Later, they found A20 promoted influenza A virus infection through impeding host antiviral response (Maelfait et al., 2012; Maelfait et al., 2016). Interestingly, Kumar and their colleagues, similarly to influenza A virus infection as we reported, the expression of let-7f decreased with the progression of Mtb infection in mice, and A20 increased. Also, let-7f inhibited Mtb infection by targeting A20, a feedback inhibitor of the NF-κB pathway. These results reveal a role for let-7f and its target A20 in regulating immune responses to Mtb and controlling bacterial burden (Kumar et al., 2015). These studies further highlight the regulatory roles of let-7 and its downstream targets in bacterial, viral and other microbial infections. Is RPS16 a key inflammatory regulator exerts cellular regulation beyond its ribosomal function? What is the specific mechanism of action in the infection of viruses, bacteria and other pathogenic microorganisms? We will continue to explore in depth in future study.

Finally, there are still some shortcomings and deficiencies in this study. We need to confirm whether let-7 has antiviral effect *in vivo*. In our research, we found that let-7 also promoted the expression of type I interferon in cells transfected with poly I:C, does let-7 have inhibitory effects on other RNA viruses other than influenza? Is the regulatory effect of let-7 on viral infection limited to RPS16, or does it also regulate viral replication through other important targets? In our previous study, we also found that knockdown of RPS16 enhanced the expression of pTBK1 after virus infection and after poly I:C stimulation of cells, what is the specific regulatory mechanism? How about the *in vivo* regulatory effect of RPS16 towards influenza and other virus infection? We will discuss in depth in future research.

In summary, we performed miRNA profiling of A549 cells during IAV infection from two microarray datasets, bioinformatics analysis revealed that two members of let-7 miRNAs (hsa-let-7b and hsa-7f-3p) were down-regulated and regulated IAV infection. However, more experiments need to be performed to confirm their biological functions. Furthermore, we found that mimics of let-7b/7f inhibited IAV infection at least partially through repressing RPS16 expression. Although this study leaves some shortcoming to be desired, the findings here may still provide a perspective on the potential use of let-7 and its downstream target RPS16 as targets for the treatment of viral infections.

## Data Availability Statement

The raw data supporting the conclusions of this article will be made available by the authors, without undue reservation.

## Author Contributions

WW, CW, and CX performed experiments. WW and SL contributed reagents, materials, and analytical tools. WW organized the data and wrote the manuscript. QM and SL supported the project and revised the manuscript accordingly. All authors have reviewed and revised the manuscript.

## Funding

This work was supported by the Guangzhou Science and Technology Plan Project, China (202102021242), the Doctoral Workstation Project of Guangdong Second Provincial General Hospital (No. 2019BSGZ012), the Science foundation of Guangdong second Provincial General Hospital (TJGC-2021008) and the Guangdong Science and Technology Plan Project (2021B1212030008).

## Conflict of Interest

The authors declare that the research was conducted in the absence of any commercial or financial relationships that could be construed as potential conflicts of interest.

## Publisher’s Note

All claims expressed in this article are solely those of the authors and do not necessarily represent those of their affiliated organizations, or those of the publisher, the editors and the reviewers. Any product that may be evaluated in this article, or claim that may be made by its manufacturer, is not guaranteed or endorsed by the publisher.

## Supplementary Material

Supplementary Figure 1 Protein synthesis and cytotoxicity caused by the cell transfected with let-7 mimics or siRPS16. (A) Protein synthesis was detected for cells transfected with let-7 mimics or siRPS16 at 48h by HPG protein synthesis assay. The cell nucleus was stained with DAPI. The rate of HPG incorporation was examined using the wavelength of 488 nm for the fluorescent intensity. (B) The fluorescence intensities of the images were obtained by counting the four fields of view per sample using image J. Data were shown as mean ± SD. (C) The flow cytometry was conducted after the cell was transfected with let-7b/f mimic (or miR NC control) and siRPS16 (or siNC control) for 48h, respectively. The cells were staining with Annexin-V/PI and analyzed by flow cytometer. (D) Population of the PI positive and negative cells of each sample to represent the proportion of dead cells and live cells. Data were shown as mean ± SD.

Supplementary Figure 2 HA and other inflammatory cytokines expression. (A) A549 cell was transfected with the mimics of let-7b/7f or siRPS16 and negative control for 48h. The influenza HA gene expression was detected by RT-PCR after the transfected cell infected with influenza A/WSN/1933 at 0.1 MOI for 24h. (B) A549 cell was transfected with the mimics of let-7b/7f or siRPS16 and negative control for 48h. The cells were infected with influenza A/WSN/1933 virus (MOI=1) for 12h, the expression of IL-6 and TNF-α were detected by RT-PCR and normalized to expression of GAPDH. Data were shown as mean ± SD, **p* < 0.05, ***p* < 0.01.
