# Supplementary figures and images for: MicroRNA let-7 Suppresses Influenza A Virus Infection by Targeting RPS16 and Enhancing Type I Interferon Response
Source: Front Cell Infect Microbiol. 2022 Jul 7;12:904775. doi: 10.3389/fcimb.2022.904775 (PMC9301362; doi:10.3389/fcimb.2022.904775)

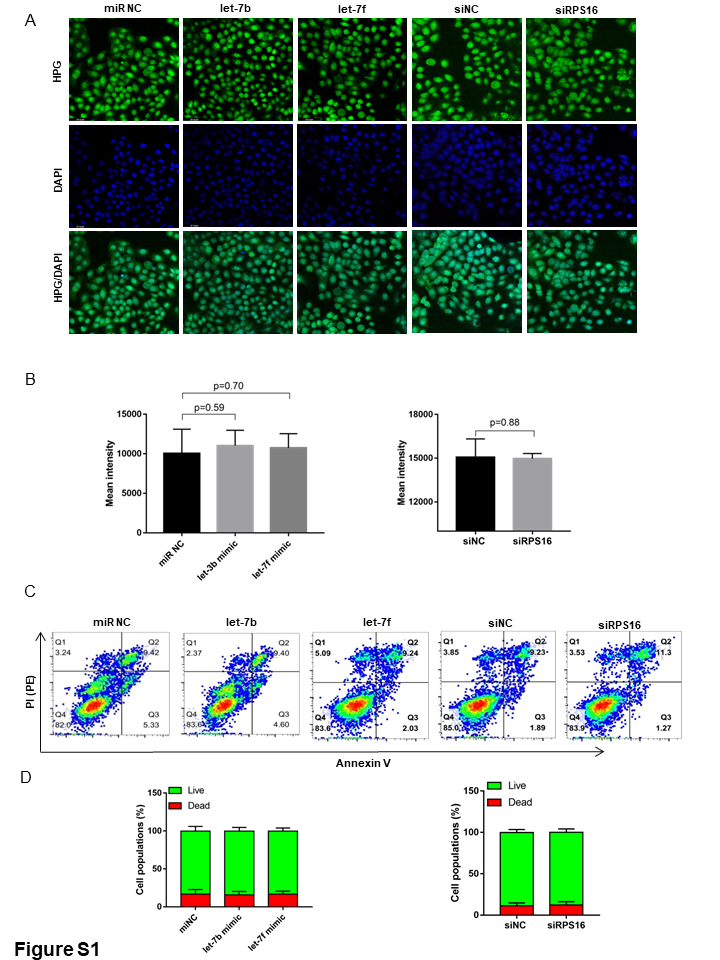

Supplement: Supplementary Figure 1 — Protein synthesis and cytotoxicity caused by the cell transfected with let-7 mimics or siRPS16. (A) Protein synthesis was detected for cells transfected with let-7 mimics or siRPS16 at 48h by HPG protein synthesis assay. The cell nucleus was stained with DAPI. The rate of HPG incorporation was examined using the wavelength of 488 nm for the fluorescent intensity. (B) The fluorescence intensities of the images were obtained by counting the four fields of view per sample using image J. Data were shown as mean ± SD. (C) The flow cytometry was conducted after the cell was transfected with let-7b/f mimic (or miR NC control) and siRPS16 (or siNC control) for 48h, respectively. The cells were staining with Annexin-V/PI and analyzed by flow cytometer. (D) Population of the PI positive and negative cells of each sample to represent the proportion of dead cells and live cells. Data were shown as mean ± SD. [file Image_1.tif]

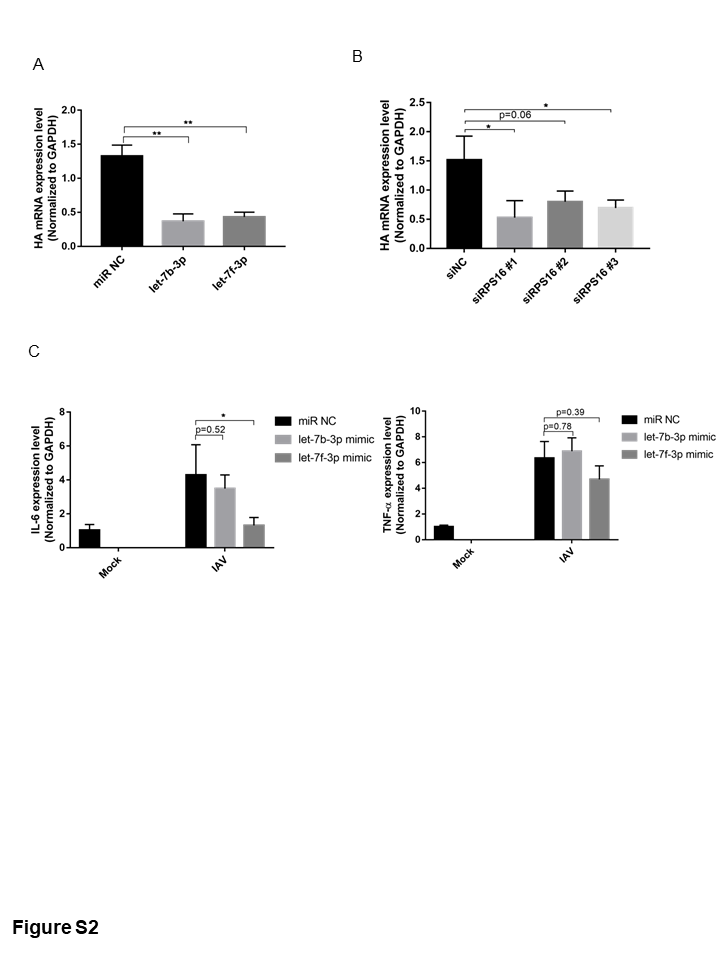

Supplement: Supplementary Figure 2 — HA and other inflammatory cytokines expression. (A) A549 cell was transfected with the mimics of let-7b/7f or siRPS16 and negative control for 48h. The influenza HA gene expression was detected by RT-PCR after the transfected cell infected with influenza A/WSN/1933 at 0.1 MOI for 24h. (B) A549 cell was transfected with the mimics of let-7b/7f or siRPS16 and negative control for 48h. The cells were infected with influenza A/WSN/1933 virus (MOI=1) for 12h, the expression of IL-6 and TNF-α were detected by RT-PCR and normalized to expression of GAPDH. Data were shown as mean ± SD, *p < 0.05, **p < 0.01. [file Image_2.tif]
